# A secondary analysis examining the concordance of self-perception of weight and actual measurement of body fat percentage: The CRONICAS Cohort Study

**DOI:** 10.1186/s40608-019-0229-5

**Published:** 2019-04-01

**Authors:** Anthony L. Bui, Miguel G. Moscoso, Antonio Bernabe-Ortiz, William Checkley, Robert H. Gilman, Liam Smeeth, J. Jaime Miranda

**Affiliations:** 1 David Geffen School of Medicine at UCLA, Los Angeles, CA USA; 2 CRONICAS Centre of Excellence in Chronic Diseases, Universidad Peruana Cayetano Heredia, Av. Armendáriz 497, Miraflores, Lima 18 Peru; 3 School of Public Health and Administration, Universidad Peruana Cayetano Heredia, Lima, Peru; 4 Department of International Health, Bloomberg School of Public Health, Johns Hopkins University, Baltimore, MD USA; 5 Faculty of Epidemiology and Population Health, London School of Hygiene and Tropical Medicine, London, UK; 6 School of Medicine, Universidad Peruana Cayetano Heredia, Lima, Peru

**Keywords:** Obesity, Weight self-perceptions, Chronic disease, Peru

## Abstract

**Background:**

Individuals’ self-perceptions of weight often differ from objective measurements of body fat. This study aimed to 1) measure agreement between self-perceptions of weight and objective measurement of body fat by bioelectric impedance analysis (BIA) among Peruvian adults; and 2) quantify the association between body fat and a) baseline self-perceptions of weight and b) whether a participant underestimated their weight status.

**Methods:**

Longitudinal data from the CRONICAS Cohort Study of 3181 Peruvian adults aged 35-years and older were used. BIA measurements of body fat were categorized across four nominal descriptions: low weight, normal, overweight, and obese. Kappa statistics were estimated to compare BIA measurements with baseline self-perceptions of weight. To quantify the association between body fat over time with both baseline self-perceptions of weight and underestimation of weight status, random effects models, controlling for socioeconomic and demographic covariates, were employed.

**Results:**

Of the 3181 participants, 1111 (34.9%) were overweight and 649 (20.4%) were obese at baseline. Agreement between self-perceived and BIA weight status was found among 43.1% of participants, while 49.9% underestimated and 6.9% overestimated their weight status. Weighted kappa statistics ranged from 0.20 to 0.31 across settings, suggesting poor agreement. Compared to perceiving oneself as normal, perceiving oneself as underweight, overweight, or obese was associated with − 4.1 (*p* < 0.001), + 5.2 (*p* < 0.001), and + 8.1 (*p* < 0.001) body fat percentage points, respectively. Underestimating one’s weight status was associated with having 2.4 (*p* < 0.001) body fat percentage points more than those not underestimating only after adjusting for demographic and socioeconomic covariates.

**Conclusions:**

Half of study participants were overweight or obese. There was poor agreement between self-perceptions of weight with BIA measurements of body fat, indicating that individuals often believe they weigh less than they actually do. Underestimating one’s weight status was associated with having more body fat percentage points, but was only statistically significant after adjusting for demographic and socioeconomic characteristics. Further research should be conducted to investigate how self-perceptions of weight can support clinical and public health interventions to curb the obesity epidemic.

## Background

The prevalence of overweight and obesity is rising globally [1, 2]. Better understanding of behavioral factors that may influence obesity, such as an individual’s self-perception of his or her weight, is necessary to address the current obesity epidemic. Poor self-perception of weight is associated with psychological stress [3], body image issues [4], and risk factors for poor health including poor diet, smoking, and alcohol consumption [5]. Through these mechanisms, self-perception of weight likely influences an individual’s health, and, in particular, objective measurement of his or her weight. For example, self-perception of weight may influence lifestyle factors that can result in changes in body fat over time. It has been found that adults who have correctly assessed that they are overweight may practice healthier dietary habits [6]. Exploring how self-perceptions of weight relate to reality may help frame the way clinicians and public health leaders think about interventions to curb obesity.

Many studies have analyzed the association between self-perceptions of weight and body mass index (BMI) and have found varying agreement or predictability between these two measurements [7–13]. One of the limitations of BMI is its inability to differentiate lean and fat body mass [14]. Percent body fat assessed by bioelectric impedance (BIA), has been found to be a strong and effective measurement of body fat [15]. Since many studies of self-perceptions of weight are cross-sectional, our understanding of the relationship between self-perceptions and objective measurements of body composition over time is limited [10–13]. Given that weight and body composition are not static, longitudinal data provide an opportunity to assess individuals’ variations in body fat prospectively over time.

This study had three objectives. First, the study aimed to measure agreement between participants’ self-perceptions of weight and weight status as defined by BIA-measured body fat percentage points in a cohort of Peruvian adults living in both urban and rural settings. The second objective was to quantify the association between body fat over time and baseline self-perceptions of weight. The final objective was to quantify the association between body fat over time and whether or not a participant underestimated his or her baseline weight status.

## Methods

### Data source

This secondary analysis employed data from the CRONICAS Cohort Study, a longitudinal data series that measured indicators related to cardiovascular health. The data includes both clinical measurements, such as bioelectric impedance analysis, as well as self-reported information obtained by questionnaires [16]. The CRONICAS Cohort Study included Peruvian adults living in four regions that varied in urbanicity and altitude: highly urbanized Lima (at sea level), urban and rural Puno (at 3825 m above sea level), and semi-urban Tumbes (at sea level) [16]. Participants of the cohort were 35-years-old or older and were able to provide consent. This analysis utilized enrollment and baseline data from the CRONICAS Cohort, which were collected in September 2010. This study also utilized data from the first two follow-up visits, which occurred an average of 15 and 30 months after participants’ baseline visit [16].

### Variables

#### Body fat and body mass index

The outcome variable of this study is body fat percentage points assessed by BIA. In the CRONICAS Cohort Study, BIA measurements were taken using a TBF-300A body composition analyzer (TANITA Corporation, Tokyo, Japan) using the manufacturer’s specifications. The device measures the subject’s body composition, including water and fat by transmitting an electrical signal through the subject, enabling it to measure resistance, or impedance, once the signal interacts with fat tissue. This impedance metric is used to calculate body composition measurements via preprogrammed proprietary Tanita equations. The device with its corresponding equations have been used and validated in other studies showing that BIA is a strong predictor of body fat [17–21]. Measurements of body fat percentage points and weight were reported from the body composition analyzer and were recorded at baseline and at two subsequent follow-up visits. All subjects were barefoot and wore light clothing when measurements were taken [16]. Clinical assessments of subjects occurred at baseline. Height measurements were taken utilizing standardized techniques. BMI data were calculated using height and weight [16].

#### Self-perceptions of weight

Weight status is categorized into four nominal groups: “low weight,” “normal,” “overweight,” and “obese.” The CRONICAS Cohort Study utilized a baseline questionnaire that asked participants to self-assess their weight and select one of the four weight categories that they thought best represented their weight at that time, providing baseline data for self-perception of weight. The questionnaire was developed through the World Health Organization’s (WHO’s) STEP approach to survey non-communicable disease [16]. Participants were not given explanations about the descriptions of the different weight categories, and they were not able to see their actual weight at baseline. They were only notified about their measurements after finishing their survey. Further information regarding the CRONICAS questionnaire can be found in the original study protocol [16].

#### Weight status definition

Both BIA measurements of body fat percentage points and BMI are numeric, continuous variables. For this analysis, both BIA measurements and BMI were categorized into the four nominal descriptions of weight status described above. BIA measurements of body fat percentage points at baseline were categorized into the four descriptive groups based on sex, age, and ethnic group-specific definitions using Gallagher’s classification [22], which uses BMI to predict and define body fat cut-off points, an approach employed in previous studies [23–25]. For males aged 35–39 years, having a maximum of 8 % body fat was categorized as underweight, having at least 21% but less than 26% was categorized as overweight, and having at least 26% was categorized as obese. Those between 8 % and 21% body fat were categorized as normal. For the following age and sex groups, the cutoffs between underweight and normal, normal and overweight, and overweight and obese were as follows: for females 35–39 years: 21, 33, and 39; for males 40–59 years: 11, 23, and 29; for females 40–59 years: 23, 35, and 41; for males 60 years and older: 13, 25, and 31; for females 60 years and older: 25, 38, and 43. Because the Gallagher’s classification did not have specifications for individuals age 80 years and above, it was assumed that this age group had the same definitions as the age 60 to 79 group, as was done in a previous study [23]. BMI were categorized into the four groups using standard definitions [26].

#### Underestimation of weight status

A binary variable indicating whether or not a participant underestimated his or her weight status was created by comparing self-perceptions of weight and BIA-defined weight categories. Participants who had a self-perceived weight category that was lower than their BIA-defined category were considered to have underestimated. In contrast, those who had a self-perceived weight category that was the same or higher than their BIA-defined category were not considered to have underestimated.

#### Time

The data in this study reports age in years, rounded to the nearest tenth of a year. Age was calculated by subtracting a participant’s visit date from his or her birth date, allowing for greater precision of the age variable across baseline and subsequent follow-up visits.

#### Socioeconomic status, sex, and education

Socioeconomic status was determined using a wealth index taken from the CRONICAS Cohort Study. The wealth index was derived from survey questions regarding possession of assets (e.g., radio, TV, and internet) and household facilities (e.g., water source, floor material, and fuel use), and then split into three evenly ranked groups (low, medium, and high) [16]. Income was not used to determine socioeconomic status. This method has been employed in epidemiological studies in other low- and middle-income countries [27]. Sex was self-reported by each participant. Education level was also obtained from a self-reported questionnaire, in which participants reported the highest number of years of education that they had obtained. Education was then categorized into three groups: less than 7 years, seven to 11 years, and 12 or more years of education.

### Data analysis

#### Measuring agreement

Two methods were used to compare BIA measurements of body fat to baseline self-perceptions of weight status. First, percentage agreement between these two metrics was calculated across all samples and then by setting. Second, both unweighted and weighted kappa statistics were estimated across all samples and then by setting. Confidence intervals at the 95th percentile were estimated using a bootstrap methodology of 1000 replications. Using the kappa statistic has been substantiated among many other studies comparing self-perceptions of weight with objective measurements of obesity [11, 28–30]. The kappa statistic is commonly used to measure agreement between two or more observers. A kappa statistic of 1 indicates perfect agreement between the two observers, while a kappa statistic of 0 indicates that agreement could have been due to chance [31]. Weighted kappa statistics weigh further discordant categories more heavily than closer ones. Percentage agreement and kappa statistics were also estimated to measure agreement between body fat with BMI and between self-perceptions of weight and BMI.

#### Quantifying the associations between body fat over time with both self-perceptions of weight and underestimation of weight status

A random effects model was employed to quantify the association of body fat over time with self-perceived weight category at baseline. Random effects regression models are often used for clustered or longitudinal data. Equation 1 lays out the model. The outcome variable, body fat as measured by BIA, is a continuous variable. The explanatory variable, self-perceptions of weight, is a categorical variable; its reference category is normal weight. The model includes an age variable that is centered at 35 years (i.e., 35 was subtracted from each age data point), which serves as a continuous variable representing time. Covariates (i.e. potential confounders) are represented as λ; these include age at baseline, sex, socioeconomic status, setting, and education level. Both crude and adjusted models were used.

Equation 2 lays out the model for the third objective of the study: to quantify the association between body fat over time and whether or not a participant underestimated his or her baseline weight status. Though it is similar to Eq. 1, Eq. 2 contains an explanatory variable that reflects whether or not a participant underestimated his or her weight status at baseline. This component of the study focused on individuals who underestimated their weight but were objectively overweight or obese. Thus, individuals who underestimated their weight status but were objectively normal were excluded from this analysis.

Robust standard errors were specified for both equations. Data were cleaned to address discrepancies in these variables, and a list-wise method was used to drop observations missing in the majority of variables of interest. Twelve percent of the original data from 3619 participants were therefore removed because they were missing variables. Data analysis was conducted using Stata 14 (StataCorp, TX, US).

1 $$ \left[{BIA}_t\right]={\beta}_0+{\beta}_1\left[ SPW\right]+{\beta}_2\left[ centered\_ age\right]+{\beta}_3\left[ SPW\ast centered\_ age\right]+{\beta}_4\left[\lambda \right] $$

2 $$ \left[{BIA}_t\right]={\beta}_0+{\beta}_1\left[ underestimate\right]+{\beta}_2\left[ centered\_ age\right]+{\beta}_3\left[ underestimate\ast centered\_ age\right]+{\beta}_4\left[\lambda \right] $$

## Results

### General characteristics

Table 1 displays characteristics of the 3181 participants in the study according to their BIA weight status. While 55.3% of the participants were overweight or obese, only 4.4% of participants were underweight. The prevalence of being overweight or obese was lower among participants who were 65-years-old or older. Among the four settings, rural Puno had the lowest rates of overweight and obese weight statuses at 31.0% of its 565 participants, compared to 63.5% in urban Puno. Across the entire study population, there were lower proportions of overweight and obese individuals in the group with lowest socioeconomic status (40.8%) compared to the middle (57.8%) and highest groups (66.4%). Similarly, there were lower proportions of overweight and obese among individuals with less than 7 years of education (45.8%) compared to those with seven to 11 years (61.2%) and 12 or more years of education (66.7%).

**Table 1:** Demographic characteristics of the study population by weight status as body fat measured by BIA

	Underweight	Normal	Overweight	Obese	Total
	*n* (%)	*n* (%)	*n* (%)	*n* (%)	*n*
Total	141 (4.4%)	1280 (40.2%)	1111 (34.9%)	649 (20.4%)	3181
Age					
35–44	11 (1.4%)	267 (34.5%)	295 (38.1%)	202 (26.1%)	775
45–54	24 (3.0%)	287 (35.3%)	322 (39.6%)	181 (22.2%)	814
55–64	31 (3.8%)	316 (39.2%)	279 (34.6%)	181 (22.4%)	807
65 +	75 (9.6%)	410 (52.2%)	215 (27.4%)	85 (10.8%)	785
Sex					
Female	122 (7.4%)	647 (39.5%)	555 (33.9%)	315 (19.2%)	1639
Male	19 (1.2%)	633 (41.1%)	556 (36.1%)	334 (21.7%)	1542
Setting					
Lima	26 (2.5%)	384 (37.1%)	399 (38.6%)	225 (21.8%)	1034
Urban Puno	18 (3.2%)	185 (33.3%)	198 (35.7%)	154 (27.8%)	555
Rural Puno	70 (12.4%)	320 (56.6%)	130 (23.0%)	45 (8.0%)	565
Tumbes	27 (2.6%)	391 (38.1%)	384 (37.4%)	225 (21.9%)	1027
Assets					
Lowest	94 (9.3%)	506 (49.9%)	282 (27.8%)	132 (13.0%)	1014
Middle	26 (2.4%)	427 (39.8%)	404 (37.6%)	217 (20.2%)	1074
Highest	21 (1.9%)	347 (31.8%)	425 (38.9%)	300 (27.5%)	1093
Education level					
< 7 years	111 (7.6%)	678 (46.6%)	454 (31.2%)	213 (14.6%)	1456
7–11 years	21 (2.0%)	382 (36.8%)	396 (38.1%)	240 (23.1%)	1039
12+ years	9 (1.3%)	219 (32.0%)	261 (38.2%)	195 (28.5%)	684

Note: Percentages are presented across rows, such that the total of a row equals 100%

### Weight status agreement and weight underestimation

Table 2 shows results of the analysis measuring agreement between self-perceptions of weight and BIA weight status in terms of percentage agreement and kappa statistics. At baseline, a total of 43.2% of the study population perceived their weight to be in a category that agreed with their BIA measured weight. This translates to a kappa statistic of 0.16. Across the four residential settings at baseline, percentage agreement and kappa statistics were highest in Tumbes (46.5% and 0.19, respectively) and lowest in Rural Puno (37.2% and 0.08). Weighted agreement and kappa statistics were highest in Lima (80.5% and 0.30) and Tumbes (79.7% and 0.31) and lowest in Rural Puno (75.8% and 0.20).

**Table 2:** Agreement, underestimation, overestimation, and kappa statistics of self-perceptions of weight compared to body fat as measured by bioelectric impedance (BIA) across residential settings at baseline

	Total	Lima	Urban Puno	Rural Puno	Tumbes
	*n* = 3181	*n* = 1034	*n* = 555	*n* = 565	*n* = 1027
Agreement	43.20%	46.30%	37.30%	37.20%	46.50%
Underestimation	49.90%	43.70%	56.80%	55.80%	49.30%
Overestimation	6.90%	10.00%	6.00%	7.10%	4.30%
Kappa (95% CI)	0.16 (0.14–0.19)	0.18 (0.14–0.22)	0.10 (0.05–0.16)	0.08 (0.02–0.13)	0.19 (0.15–0.23)
Weighted agreement	78.70%	80.50%	76.50%	75.80%	79.70%
Weighted kappa (95% CI)	0.31 (0.29–0.33)	0.30 (0.27–0.34)	0.27 (0.23–0.31)	0.20 (0.15–0.25)	0.31 (0.27–0.34)

Note: Confidence intervals for kappa coefficients were estimated using a bootstrap methodology of 1000 replications

**Fig. 1:**
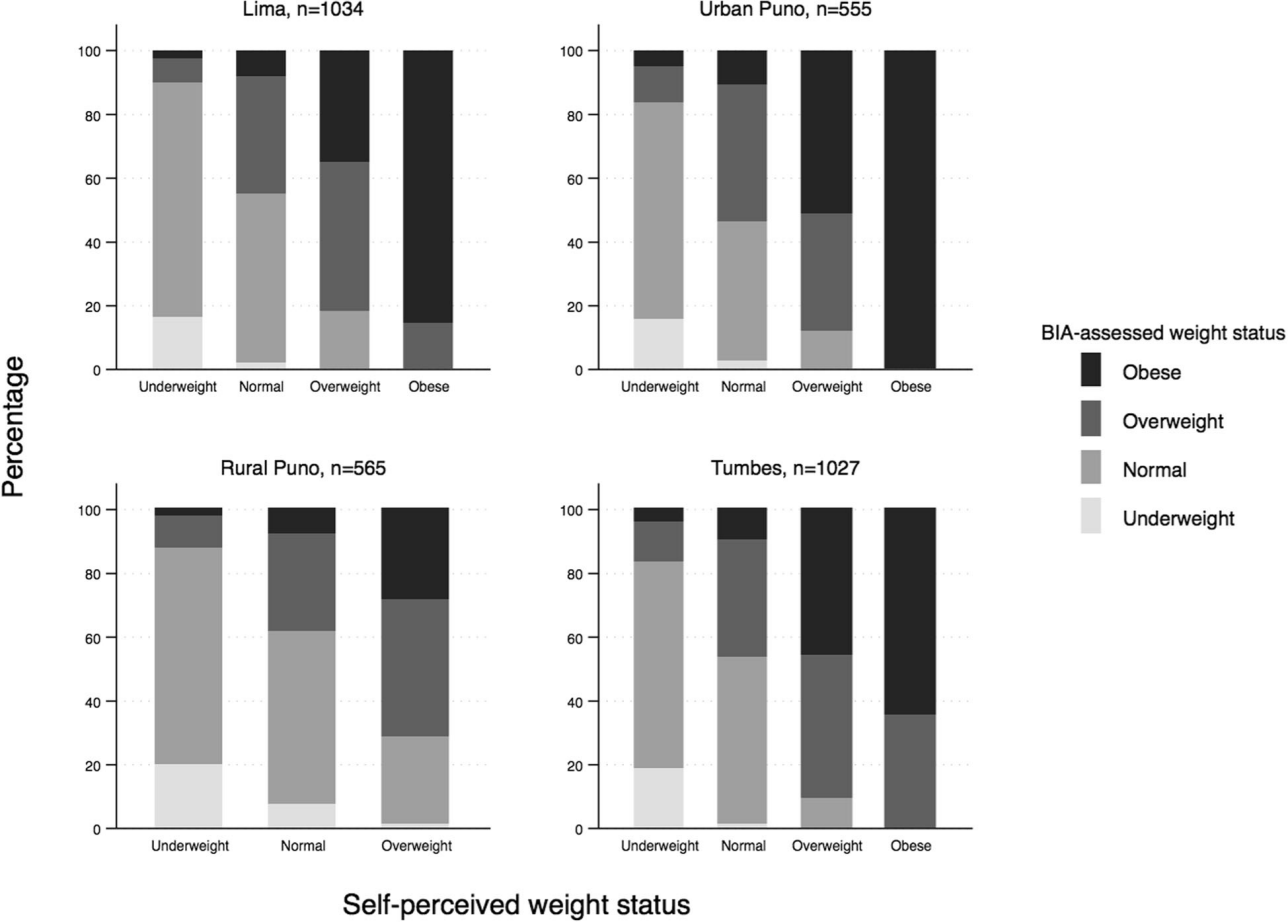
Self-perceptions of weight vs. body fat as measured by bioelectric impedance (BIA) across residential setting Note: No participants in Rural Puno self-perceived to be obese. Each of the four panels reports a different residential setting in Peru. For each setting, four bars represent the four different self-perceptions of weight groups. The stacks within the bars represent the percentage breakdown of objective weight status assessed by bioelectric impedance analysis (BIA) within each self-perception of weight group

While 43.2% of participants’ self-perceived weight agreed with their BIA weight status, 49.9% underestimated and 6.9% overestimated their weight status. Among the 1371 participants whose self-perceptions of weight agreed with their BIA weight status, 98 (7.1%) were underweight, 745 (54.3%) were normal, 510 (37.1%) were overweight, and 20 (1.5%) were obese. The proportion of those who underestimated their weight status ranged from 43.7% to 56.8% across the four residential settings. Among those who underestimated, 540 participants, representing 17.0% of all study participants, perceived their weight to be normal while their BIA weight status was overweight. Figure 1 displays bar graphs depicting the comparison of self-perceptions of weight status and BIA weight status, showing agreement, underestimation, and overestimation of weight across the four residential settings. Participants who perceived their weight to be obese tended to be objectively obese. In rural Puno, no participants perceived their weight to be obese, though some were objectively categorized as obese from their BIA weight status.

In terms of agreement between self-perceptions of weight and BMI, 33.1% of participants had accurate self-perceptions of their weight status. In contrast, 65.7% of participants underestimated their weight status in comparison to BMI. In terms of the two objective measurements of weight status, 62.8% of participants had BMI and BIA measurements that fell into the same weight category. Meanwhile, 30.9% of participants were placed in a higher weight status by BMI than by BIA measurement.

### Association of self-perceptions of weight and body fat

Table 3 shows the association between self-perceptions of weight and body fat over time. The crude model shows that participants who perceived themselves to be underweight had, on average, 3.1 (*p* < 0.001) less body fat percentage points than participants who perceived their weight to be normal. In contrast, participants who perceived themselves to be overweight or obese had, on average, 7.2 (*p* < 0.001) and 10.7 (*p* < 0.001) more body fat percentage points than participants who perceived their weight to be normal, respectively. As age increased, participants gained 0.05 (*p* < 0.001) body fat percentage points each year. The increase in body fat percentage points was not statistically significant across categories of self-perceived weight. After adjusting for age, sex, residential setting, socioeconomic status, and education level, results show that participants gained 0.03 (*p* < 0.01) percentage points of body fat each year.

**Table 3:** Association of baseline self-perceptions of weight on body fat over time

	Crude Model		Adjusted Model	
	Coefficient	95% CI	Coefficient	95% CI
Self-perceptions of weight				
Underweight	−3.114***	−4.61, − 1.62	−4.132***	−5.29, − 2.97
Overweight	7.212***	6.19, 8.24	5.186***	4.43, 5.94
Obese	10.747***	6.11, 15.39	8.132***	4.89, 11.37
Centered Age (at 35 years)	0.053***	0.02, 0.08	0.031**	0.01, 0.05
Underweight * Centered Age	−0.011	−0.07, 0.04	− 0.011	− 0.06, 0.03
Overweight * Centered Age	0.03	−0.01, 0.07	0.031	0.00, 0.06
Obese * Centered Age	0.179	−0.13, 0.49	0.182	−0.02, 0.39
Constant	27.223***	26.51, 27.94	22.303***	21.46, 23.15

* *p* < 0.05, ** *p* < 0.01, *** *p* < 0.001

### Association of underestimation and body fat

Table 4 shows the association between underestimation of one’s weight and body fat over time. Crude models show that those who underestimated their weight category tended to have 0.7 (*p* > 0.05) more body fat percentage points than those who correctly estimated or overestimated their weight status. However, this finding was not statistically significant. Adjustment for age, sex, residential setting, socioeconomic status, and education level increased the effect size of the underestimation variable from 0.7 (*p* > 0.05) to 2.4 (*p* < 0.001) body fat percentage points.

**Table 4:** Association of baseline underestimation of weight on body fat over time as centered age

	Crude Model		Adjusted Model	
	Coefficient	95% CI	Coefficient	95% CI
Underestimate	0.723	−0.58, 2.03	2.441***	1.51, 3.37
Centered Age	0.017	−0.03, 0.07	0.001	−0.04, 0.05
Underestimate * Centered Age	0.024	−0.04, 0.09	0.04	−0.01, 0.09
Constant	33.462***	32.41, 34.51	26.010***	24.89, 27.13

* *p* < 0.05, ** *p* < 0.01, *** *p* < 0.001

## Discussion

### Key findings

More than half of Peruvian adults in this study are overweight and obese, consistent with previously reported prevalence rates of overweight and obesity in Peru [2]. Furthermore, self-perceptions of weight were found to poorly agree with objective measurements of weight, as measured by both BIA and BMI. Among all participants, the agreement with self-perceptions of weight was found to be 43.2% for BIA weight status and 33.1% for BMI. More than half of the study population underestimated its weight status.

While comparing BIA weight status and BMI was not the intention of this study, these findings suggest that BIA may have less stringent definitions of overweight and obesity. One explanation for these differences is that BIA weight statuses are specific to age and sex groups while BMI is not. Nevertheless, further research is needed to investigate why BMI and BIA differ in regards to their relationship with self-perceptions of weight. The two objective measurements of weight status, BMI and BIA, have greater kappa statistics and greater percentage agreement with each other, suggesting that perceptions are less closely aligned with objective measurements. Overall, these findings suggest that individuals poorly assess their own weight status when compared to either objective measurement.

Agreement between self-perceptions of weight and BIA weight status varied across residential setting. Participants that reside in both rural and urban Puno had lower agreement (37.2 and 37.3%, respectively), compared to Lima and Tumbes (46.3 and 46.5%). Further research should be conducted to understand how geographic and cultural factors may influence self-perceived weight in these communities.

Despite poor agreement between self-perceptions of weight and BIA weight status, this study found that self-perceptions of weight were closely associated with continuous measurements of body fat percentage points. Perceiving oneself as underweight was associated with having less body fat percentage points compared to those who perceive themselves as normal, while those who perceive themselves to be overweight and obese had higher body fat. This suggests that while there is poor self awareness of one’s own weight group, those with more body fat may tend to place themselves in the higher weight categories. Similarly, those with less body fat may tend to place themselves in the lower weight categories. However, this is also likely influenced by the fact that nearly half of the study participants did not underestimate their weight status.

Furthermore, this study explores the relationship between underestimating one’s weight status and objective measurements of body fat over time. Crude analysis suggested no statistically significant difference in body fat between those who underestimate their weight status and those who do not. However, after controlling for age, sex, residential setting, socioeconomic status, and education level, those who underestimate their weight status tend to have 2.4 more body fat percentage points than those who do not. This suggests a positive association between weight underestimation and body fat percentage points.

### Comparison with literature

Many studies have found discordance between self-perceptions of weight and objective measurements of body fat [7–13]. This study provides five major contributions to the literature on this topic. First, this study focuses on BIA, rather than BMI, as an objective measurement of body fat. Second, this study is set in Peru. While there are several studies about obesity in the United States and Europe, there are limited studies in settings like Peru, an upper middle-income country with great socioeconomic heterogeneity and a wide diversity of geographies. Third, it utilizes a longitudinal analysis to explore the trajectory of participants’ fat statuses over time given their baseline self-perception of weight. This type of analysis results in less variation across the outcome variables, because there are multiple observations for each participant. For example, this study found that, on average, participants gained just 0.03 body fat percentage points each year and such increase was not statistically significant across categories of self-perceived weight. Fourth, this study quantifies the association between underestimating one’s weight and body fat percentage points. Past research in Peru has shown that an individual’s income, education, sex, and area of residence can influence the risk of being overweight [32]. While this study controlled for age, sex, income, and education, these factors were not the focus of this study. They should be further explored to understand the determinants of discordance between self-perception of weight and objective weight measurements. A final benefit of this study is its ability to capture the natural discrepancy between self-perceived weight and objective measurements of weight. Therefore, no efforts were made to modify the understanding or education of participants, as the intent of this study was not to evaluate the intervention of health education on self-perceptions of weight. Similarly, no efforts were made to modify the usual behavior of participants, as the intent of this study was not to distort how often participants weighed themselves. As a result, it is not certain if participants had a sense of their weight at the time of this study.

### Public health and clinical implications

This study shows that the majority of participants perceive themselves to belong to a weight group that differs from their objective weight group. Prior research indicate that this discordance may begin early in life and continue into adulthood, suggesting the possibility of long-term maintenance of discordant patterns of self-perceptions of weight [33]. According to the Transtheoretical Model and Precaution Adoption Process Model, behavior change takes place across six stages: pre-contemplation, contemplation, preparation, action, maintenance, and termination [34, 35]. Recognition of one’s weight is a critical stage in behavior change. Incorrectly assessing one’s weight may preclude healthy behaviors, such as improving one’s diet or engaging in physical activity, both of which have positive effects on weight and health in general [36]. In addition, the Social-Cognitive Theory is often applied in interventions targeting weight management and physical activity. The Social-Cognitive Theory states that individuals must utilize their self-efficacy, or resiliency, to understand and overcome the barriers to making changes and meeting their goals [37]. Further research is required to ascertain the clinical significance of using self-perception of weight to facilitate lifestyle modifications. For example, it would be worthwhile to investigate if asking about self-perception of weight during clinical encounters can help improve physician-patient communication and understanding about one’s weight. This may help improve weight management goals by aligning an individual’s perceptions with the reality of his or her weight status. While this study shows that underestimating one’s weight status may be associated with having more body fat, after controlling for demographic and socioeconomic characteristics, future research is needed to definitively understand the factors that influence an individual’s likelihood of improperly self-perceiving their weight status, as well as the factors that would motivate behavior change. Further research is also needed to assess if clinicians and public health practitioners would benefit from tailoring interventions according to self-perceptions of weight status, underestimating one’s weight status, or BIA.

### Limitations

This study has several limitations. First, the residential sites in the study included four areas that are not representative of all of Peru. Nevertheless, the sites provide a heterogeneity of settings that vary in environmental characteristics, making these results more generalizable than a study conducted at a single site. Secondly, the measurement of BIA body fat percentage points is calculated using an algorithm from TBF-300A that is proprietary to the manufacturer. While the robustness of TBF-300A results has been demonstrated in past published studies [17–20], there is no substitute for having a complete understanding of equations used to calculate BIA estimates. Still, any potential measurement error is assumed to be constant across the whole study population. In addition, BIA measurements themselves have several limitations. BIA measurements may vary depending upon several factors including consumption of food and drink, proximity of time since physical activity, temperature, and menstrual cycles [15]. These factors were not controlled for in the study and therefore may distort BIA findings. Finally, the categorization of weight status using Gallagher’s classifications may not adequately represent the Peruvian population. The original study to obtain these classifications was performed in the United Kingdom, United States, and Japan on white, African American, and Asian individuals [22]. The intention of this study was not to establish a set of new definitions of body status, but instead to utilize a mechanism to compare self-perception of weight and actual weight status. Future research to develop robust, standardized definitions of weight status using body fat is critical to linking biological and behavioral characteristics in the study of obesity.

## Conclusion

Among Peruvian adults, poor agreement between self-perceptions of weight and objective measurements of body fat using BIA was found. Longitudinal regression analysis reveals that participants in this study gained an average of 0.03 body fat percentage points each year. More than half of participants in the study underestimated their weight. After adjusting for sex, age, socioeconomic status, education and residential setting, underestimation of weight status was associated with having a greater percentage of body fat compared to not underestimating one’s weight. Future research should explore the characteristics that influence an individual to underestimate his or her weight status. Further research should also investigate how using self-perceptions of weight can impact lifestyle modifications and behavior change in clinical and public health settings.

## Abbreviations

BIA: Bioelectric impedance analysis
BMI: Body mass index
